# Alignment and contribution of nursing doctoral programs to achieve the sustainable development goals

**DOI:** 10.1186/s12960-020-00530-7

**Published:** 2020-11-07

**Authors:** Isabel Amélia Costa Mendes, Carla Aparecida Arena Ventura, Ítalo Rodolfo Silva, Elucir Gir, Emerson Willian Santos de Almeida, Artur Acelino Francisco Luz Nunes Queiroz, Bruna Sordi Carrara, Raquel Helena Hernandez Fernandes, Tiago Privado da Silva, Álvaro Francisco Lopes de Sousa

**Affiliations:** 1 Ribeirão Preto College of Nursing, WHO Collaborating Centre for Nursing Research Development, University of São Paulo, Avenida dos Bandeirantes, 3900, Campus Universitário, Bairro Monte Alegre, Ribeirão Preto, SP 14040-902 Brazil; 2 Federal University of Rio de Janeiro, Rio de Janeiro, Brazil; 3 Global Health and Tropical Medicine, Instituto de Higiene e Medicina Tropical, Universidade Nova de Lisboa, Lisbon, Portugal

**Keywords:** Nurse, Nursing doctoral programs, 2030 agenda, Sustainable development goals, Global health, planetary health

## Abstract

**Background:**

Different social segments from several regions of the world face challenges in order to achieve the sustainable development goals (SDGs). Nursing represents the greatest number of health workforce in the globe, dealing with these challenges in different paths, among them the training of human resources. In this context, the goal of this study was to compare the relationship between the objectives and research areas underlying nursing doctoral programs in Latin America and the SDGs.

**Method:**

Documental research comparing data of all Latin American nursing doctoral programs and the SDGs, conducted between January and March 2020.

**Results:**

From the total of 56 existing programs in Latin America, this study analyzed 52 of them, representing 92.8% of the total. Most nursing doctoral programs have contributed to SDG 3, in addition to goals 1, 2, 4, 5, 6, 8, 9, 10, 12 and 16. The SDGs 11, 13, 14, 15 and 17 were not related to any of the analyzed programs. Data reveal that the training of nursing PhDs is essential to fulfilling these goals. Results also indicate a need of programs to remain committed to relationships that enhance nursing skills to cope with the current challenges in terms of global health, such as investments for the reduction of social and gender inequities.

**Conclusion:**

The doctoral training of nurses in Latin America needs to be better aligned with the sustainable development goals (SDGs), since there is a high concentration in SDG 3. We believe that nursing will bring a greater contribution to the movement to protect planetary health as the principles governing nursing practices are better aligned with international health demands and agendas.

## Background

Much has been discussed on how the training of PhDs in nursing may benefit the health field in order to meet the needs of the job market, and also the science and health policy fields.

Most studies report challenges and acknowledge the need for many other initiatives, both to advance nursing practice and research purposes [1–3]. Comparisons conducted in native English speaking countries in three continents converge in points regarding the need for training PhDs in the nursing field [4]. There are, however, few initiatives in terms of studies focusing on nursing and the training of PhDs in Latin America. Having PhDs in the nursing field means having the potential to deliver quality health care, facilitating access of the population to health services, and minimizing social inequalities that characterize the region.

Therefore, efforts have been made in Latin America to ensure that a critical mass of nurses leads research development in the field, with results that favor leadership and participation in public policies to impact changes in the services in alignment with the population’s health needs. One of the most effective paths has been the implementation of nursing doctoral programs in line with local and global demands [5–9].

Despite the diversity and different types and structures of nursing doctoral programs [3], workers with a doctoral degree are expected to serve as leaders in the most different spheres of practice, having in mind the interdependence of social determinants of health and global health conditions [10–17]. In this sense, PhD nurses would be more prepared to strengthen policies and strategies to collaborate with the movement to protect planetary health. During the forthcoming 10 years to achieve the objectives of the 2030 Agenda, leaders are expected to prepare the new generation of nurses under the light of the Nursing Now campaign, promoted under the auspices of the British Parliament in coordination with the World Health Organization and the International Council of Nurses [18]. For these nursing leaders to tackle the main challenges imposed on the development of nations, they need to understand and act upon global health demands considering the international agenda that proposes coordinated actions [14]. The World Health Organization recognized the contribution of nurses and midwives to patients and health systems declaring 2020 the International Year of the Nurse and the Midwife.

In this context, it is important to prepare nursing PhDs to strengthen policies and strategies that promote nursing competencies to meet global health needs. Therefore, the training of these professionals should be consonant, while they may, at the same time, directly and indirectly impact the fulfillment of the SDGs.

Therefore, training provided by nursing doctoral programs, which fosters leadership perspectives for the entire profession, should establish connections with the challenges related to the fulfillment of the SDGs, considering competencies needed to recognize the factors that determine the health of people, as well as existing social inequalities. From this perspective, this study’s objective was to compare the relationships between research areas and the objectives underlying nursing doctoral programs in Latin America and the SDGs.

## Method

Documental research [19]: data analysis resulted from the analysis of official documents, of public domain, containing information about the research areas and objectives of the nursing doctoral programs of different Latin American countries. The data were captured through the official websites of these programs.

The description of the research areas and objectives of the programs constituted the raw data. These data were codified based on the dimensions identified for the capacity building of nursing PhD: areas of knowledge, working areas, interventions, principles and aims. Preliminary codes emerged from the analysis of raw data.

Therefore, this stage of analysis aimed at expanding the descriptive capacity of the investigated phenomenon, in which the information from the raw data could generate more than one preliminary code, in cases that it had different nuances, outcomes or procedures. In sum, in this phase of data expansion, each possibility could generate more than one preliminary code.

Each preliminary code received an alphanumeric identifier that corresponded to that country. In the case of Brazil, considering the number of nursing PhD programs, the region of each program was identified, with a respective number. This method was used to facilitate finding codes.

Raw data, that is, the description of the programs’ objectives and research areas were organized on a three-column spreadsheet. The left column comprised raw data and the middle column comprised the preliminary codes that resulted from coding raw data. The third column was used to identify the SDGs related to each preliminary code.

In order to match the preliminary codes with the SDGs, the researchers listed the 17 SDGs, with a brief description of their content. This description enabled the analysis of the interrelations between the code and the SDG. This stage of the research was performed by three independent researchers and, in case of disagreement among them, another researcher from the study was included in this process.

After identifying all the SDGs that corresponded to preliminary codes, we regrouped codes according to SDGs. Then, we performed a comparative analysis between the preliminary codes of each SDG in order to generate an analytical category and its interfaces with the related SDG. Thus, all the codes related to one SDG formed a category.

This process enabled identifying the breadth of potential elements that establish connections between the training of nursing PhDs, based on the programs’ objectives and research areas and the SDGs. Descriptive analysis was performed using SPSS version 22.0.

**Fig. 1:**
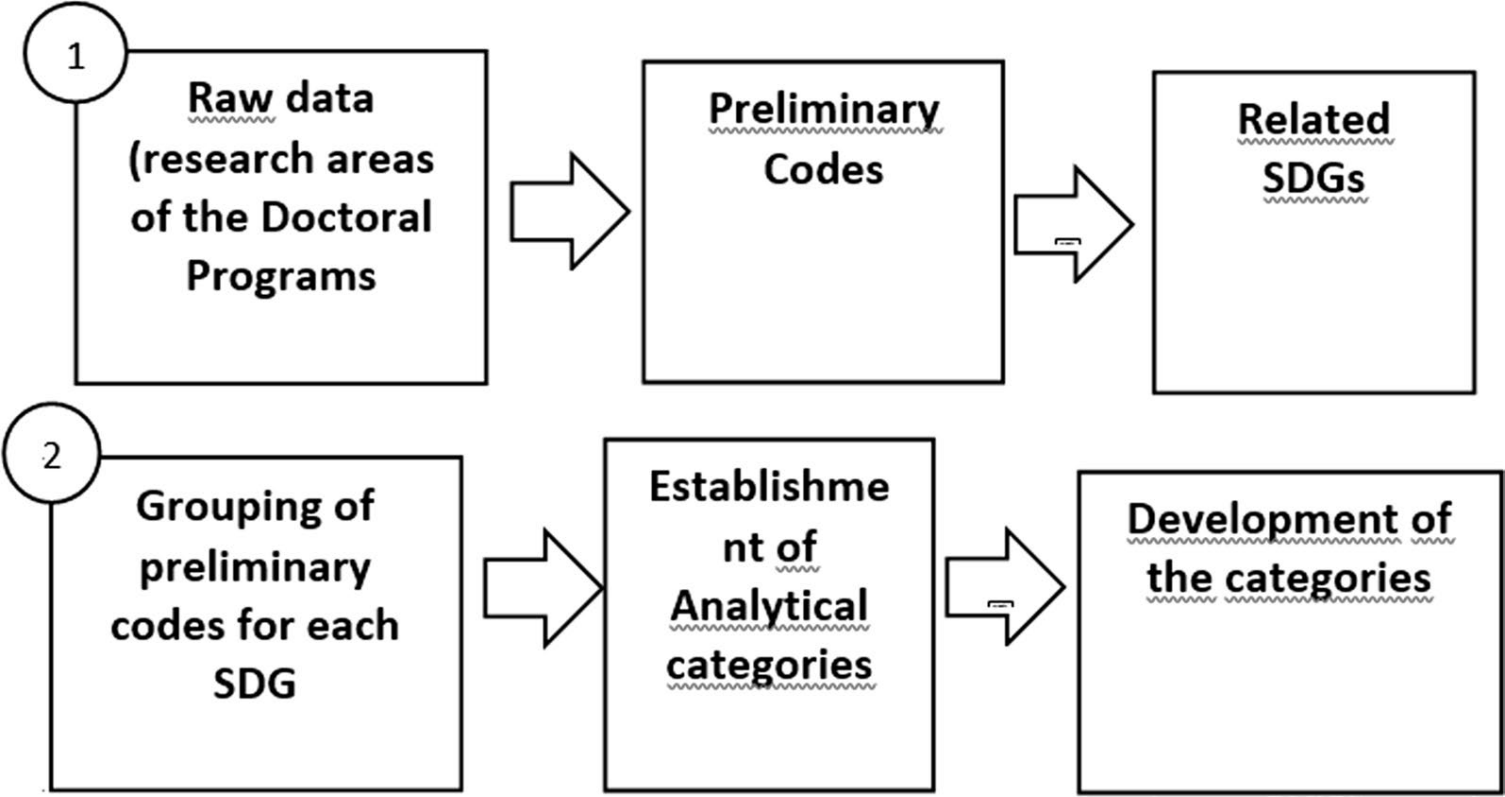
Strategy of data collection, organization, and analysis, January to March 2020

Figure 1 illustrates the process of data organization and analysis.

Data were collected from January to March 2020. The inclusion criterion was nursing doctoral programs in Latin America with data available in the internet through website. Programs that did not have online information or did not allow free access to information concerning their lines of research and objectives were excluded. Puerto Rico and French Guyana were not included in this study, as they are considered territories of other Nation States. Fifty-two out of 56 nursing doctoral programs, composed this study's sample. The analysis was conducted by independent researchers. Because data are presented in official public domain websites, approval by an Institutional Review Board is not required.

## Results

The 52 nursing doctoral programs analyzed are located in the following countries: Argentina (2), Bolivia (1), Brazil (39), Chile (2), Colombia (2), Cuba (1), Mexico (2), Panama (1), Peru (1) and Venezuela (1) (Table 1).

Data analysis enabled establishing 599 preliminary codes. After comparing and relating them with the SDGs, eight categories, which are related to 11 of the 17 SDGs, emerged. Table 2 presents the categories and the respective SDGs.

**Table 1:** Distribution of nursing doctoral programs according to country, institution and website. 2020

Institution	Site
Argentina—2	
Universidad Nacional de Tucumán	https://www.eue.unt.edu.ar/index.php/oferta-academica/doctorado-en-enfermeria.html
Universidad Nacional de Rosario (Provincia de Santa Fe)	https://fcm.unr.edu.ar/graduados-doctorados/
Bolívia—1	
Universidad Autónoma "Tomás Frías"	https://www.uatfpostgrado.edu.bo/doctorado.php
Brasil—39	
Considering the quantity of programs (39), the complete list can be found in the website of the Regulatory Agency for Doctoral Programs—CAPES (side column)	https://sucupira.capes.gov.br/sucupira/public/consultas/coleta/programa/quantitativos/quantitativoIes.jsf?areaAvaliacao=20&areaConhecimento=40400000
Chile—2	
La Facultad de Enfermería de la Universidad de Concepción	https://www.udec.cl/panoramaweb2016/content/doctorado-de-enfermer%C3%ADa-inici%C3%B3-a%C3%B1o-acad%C3%A9mico
Universidad Andrés Bello	https://investigacion.unab.cl/doctorados/doctorado-ciencia-enfermeria/
Colômbia—2	
Universidad Nacional de Colombia	https://enfermeria.bogota.unal.edu.co/menu-principal/programas/doctorados/doctorado-en-enfermeria/informacion-sobre-el-programa/
Universidad de Antioquia de Medellín	https://www.udea.edu.co/wps/portal/udea/web/inicio/estudiar-udea/quiero-estudiar-udea/posgrado/oferta-programas-posgrado/doctorados
Cuba—1	
Universidad de Ciencias Médicas de la Habana	https://instituciones.sld.cu/faenflidiadoce/banco-de-problemas/
México—2	
Facultad de Enfermería de la Universidad Autónoma de Nuevo León doctorado	https://enfermeria.uanl.mx/oferta-educativa/doctorado-en-ciencias-de-enfermeria/
Universidad de Guanajuato (UG), Campus Celaya-Salvatierra, División de Ciencias de la Salud e Ingenierías (CCS-DCSI /UG)	https://www.celayasalvatierra.ugto.mx/index.php/doctorado-en-ciencias-de-enfermeria
Panamá—1	
Facultad de Enfermería de la Universidad de Panamá	https://facenfermeria.up.ac.pa/posgrados
Perú—1	
Facultad de Enfermeria-UNT	https://www.facenf.unitru.edu.pe/index.php/facultad/plan-estrategico
VENEZUELA—1	
la Escuela de Enfermería de la Facultad de Ciencias de la Salud de la Universidad de Carabobo	https://www.postgradofcs.uc.edu.ve/doctoradoenfermeria.html

**Table 2:** Relationship between the research areas identified in the 52 nursing doctoral programs in Latin America and the sustainable development goals (SDGs), January to March 2020

Category	SDG	N	%
Nursing: implications for decreasing social inequities	SDG 1—no poverty	16	2.6
	SDG 2—zero hunger and sustainable agriculture		
Nursing science and praxis: impacts on health, economy, and quality of life of individuals and groups	SDG 3—good health and well-being	366	61.1
Education for providing necessary training to face social challenges imposed on nursing and health	SDG 4—quality education	43	7.1
Strategies to tackling social inequities from a gender perspective: nursing and women’s empowerment	SDG 5—gender equality	10	1.6
Ecological systemic care: nursing ontological and epistemological bases for intervening in contexts from a sustainable perspective	SDG 6—clean water and sanitation	17	2.8
	SDG 12—responsible consumption and production		
Labor and health: nursing implications for the healthy development of labor and workers	SDG 8—decent work and economic growth	42	7.0
Technologies and innovations for nursing and health care	SDG 9—industry, innovation, and infrastructure	35	5.8
Social nursing practice: reducing inequalities and promoting conditions for social justice	SDG 10—reduced inequalities	60	10.0
	SDG 16—peace, justice and strong institutions		
Total		599	100

The SDGs listed here show the programs are more inclined towards SDG 3—health and well-being, as a result of an affinity with the programs’ objectives and research areas, representing 61.1% (366 codes). The category “Strategies to tackling social inequities from a gender perspective: nursing and women’s empowerment” related to SDG 5—gender equality, was little represented, with only 1.6% of the preliminary codes. Note that the programs’ objectives and missions were not related to SDGs 7, 11, 13, 14 and 15. Therefore, there is still an imbalance regarding the alignment of the research areas of graduate programs with other SDGs than the SDG3.

### Nursing: implications for decreasing social inequities

This category was based on 16 (2.6%) preliminary codes and is related to SDGs 1 and 2. Data indirectly show that the training of nursing PhDs of excellence can contribute to strategies that enable interdisciplinary and intersectoral measures to combat poverty and hunger. In this sense, nursing reveals itself as a social practice aligned with the needs of populations.

This category refers to SDGs 1 and 2 considering the items: socio-cultural, political and economic determinants that influence the individuals' well-being and quality of life; relationships between society, health, and nursing; society and health production; connections between the State, society, public policies and nursing.

### Nursing science and praxis: impacts on health, economy, and quality of life of individuals and groups

This category was based on 366 (61.1%) preliminary codes. Its strong relationship with a single SDG, which is related to health, occurs because it is inserted in an important field of interest of the Nursing discipline and profession. SDG 3 is intended to promote a healthy lifestyle and well-being for individuals of all ages.

This category addresses the complexity that constitutes the contributions of nursing in its different contexts of knowledge and interventions. In order to cover its entire dimension, the category is structured into five subcategories: Human groups and families; Organizational sphere for Nursing practice; Nursing practice settings; Ontological, deontological and philosophical foundations for nursing in the field of social practice for health; nursing in the face of determinants and intervenient factors of illnesses and epidemiology.

Integrating these subcategories enables developing competencies and strategies to cope with global challenges in the health field while revealing the importance of epidemiological and intervention studies and family-centered approaches; organizational dynamics of the work process and tools to develop health care; and ethical commitment to meet social demands of health and care.

### Education for providing necessary training to face social challenges imposed on nursing and health

**Fig. 2:**
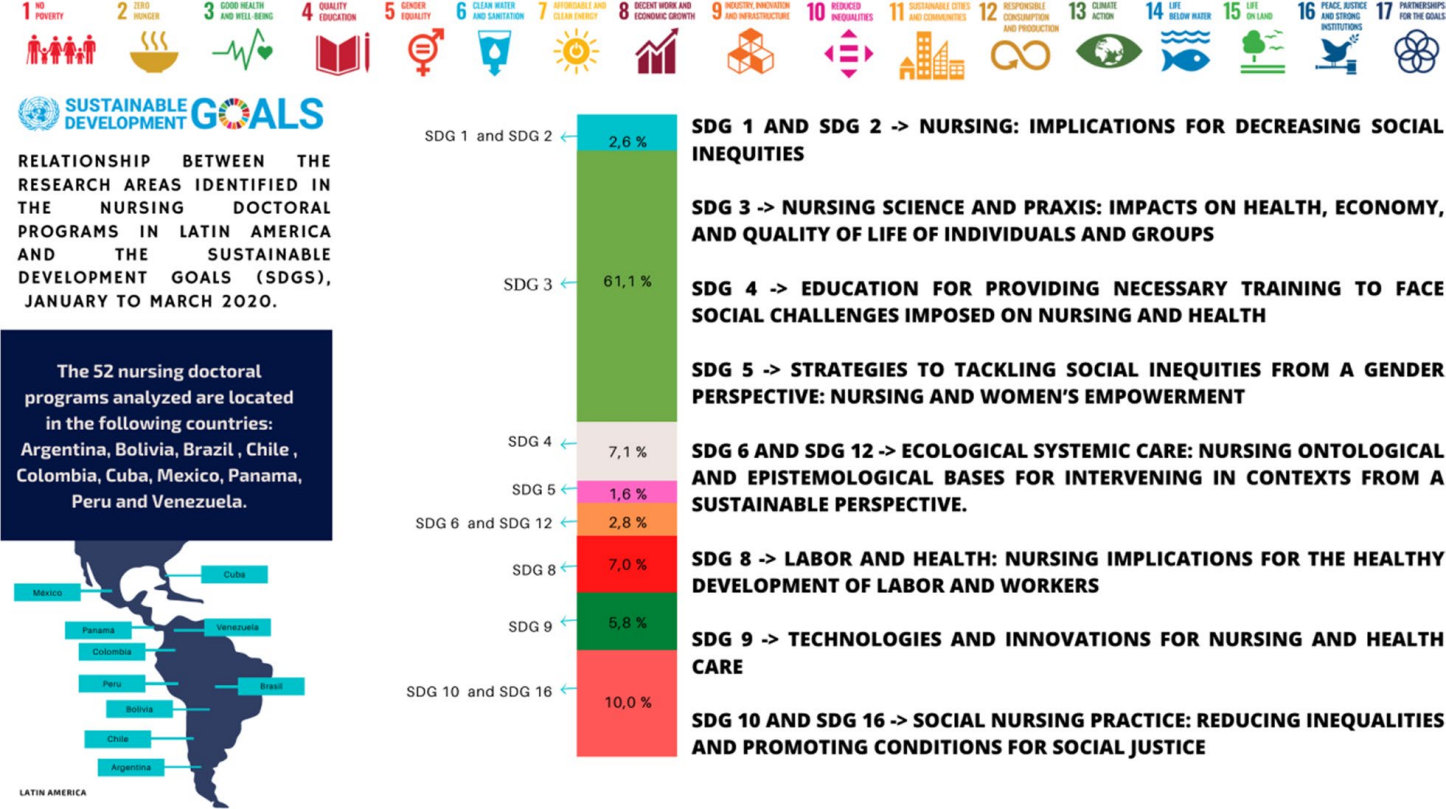
Relationship between the research areas identified in the 52 nursing doctoral programs in Latin America and the sustainable development goals (SDGs), January to March 2020

This category was based on 43 (7.1%) preliminary codes and is related to SDG 4. It addresses education elements related to the training of high school, technical, and bachelor levels of education; teaching and practitioner qualification (Fig. 2). Indirectly, in an interdisciplinary and intersectoral relationship, it reveals how nursing can contribute, with research and practice, to the quality of education provided to children and adolescents, showing there is an understanding of the importance of health for the teaching–learning process.

### Strategies to tackling social inequities from a gender perspective: nursing and women’s empowerment

This category was based on 10 (1.6%) preliminary codes and is related to SDG 5, which is intended to achieve gender equality and empower women and girls.

In this sense, nursing emerges as the first profession that requires a bachelor's degree in Latin America with a predominantly female profile. As part of the training of nursing PhDs, this category remains committed to decreasing situations that prevent women and girls from enjoying their rights and at the same time, expanding fair conditions in the job market. These discussions permeate other spheres intended to reduce inequities, which affect the specificities of women in this context, such as race, gender, and social class. Additionally, this category reveals issues concerned with the structures of societies; impacts in the health, political and economic fields, addressing gender issues from a broad and contextualized perspective.

### Ecological systemic care: nursing ontological and epistemological bases for intervening in contexts from a sustainable perspective

This category was based on 17 (2.8%) preliminary codes and is related to SDGs 6 and 12.

From Florence Nightingale’s Environmental Theory to the current developments in nursing in the fields of knowledge and professional practice, nurses have made efforts to develop studies and strategies to achieve ecological care from a complex perspective, considering interdependent connected living systems for the health of all. Hence, this category is supported on sustainability concepts; ecological care; the relationship among people/society/environment; socioeconomic, biological, environmental and clinical determinants related to the health disease-care continuum; epidemiology, biological, political–socioeconomic and cultural aspects; and tools to promote health considering improved environmental conditions.

Labor and health: nursing implications for the healthy development of labor and workers. Based on 42 (7.0%) preliminary codes, this category is related to SDG 8. Nurses design actions that enable improved conditions for work and workers substantially based on updated sound scientific knowledge. These actions impact quality, but also the economic interests of nations, as they enable to maintain the economy based on workers’ improved quality of life.

This category addresses the production of work as a social dynamics permeated by challenges that may trigger or speed up the individuals' health–disease continuum, revealing occupational risks and work-related diseases.

Technologies and innovations for nursing and health care. Based on 35 (5.8%) preliminary codes, this category is related to SDG 9. It presents a broadened perspective for the related SDG because it goes beyond inclusive industrialization, as it corroborates understanding of innovations and technologies in an inseparable logic of contemporary professions, not different from nursing and in a broad context of health. It reveals the ability of the nursing profession to create, develop and implement technologies for health care.

### Social nursing practice: decreasing inequalities and promoting conditions for social justice

This category is based on 60 (10.0%) preliminary codes and is related to SDGs 10 and 16. It reveals a perspective of citizenship based on the access and accessibility to rights and services that ensure human dignity in the face of social structuring inequities of modern society. It addresses the following: public health policies; social nursing practice; building citizenship; structure of societies; the role of the state; the role of nursing; the importance of global and universal health systems; right to health and vulnerabilities.

## Discussion

The category “Nursing: implications for decreasing social inequities" reflects the importance of training PhDs to have a leading role in the delivery of healthcare, to ensure universal health coverage and access to health care, with potential to provide a meaningful contribution to global health and achieve the sustainable development goals (SDG) [16].

Nurses play a vital role in the advancement of various aspects of SGDs in different contexts, among individuals and families, at the national and transnational contexts, from the local to the global level [20]. The challenges facing the planet and our own communities have major implications for nursing and nurses, and shared our hope that nurses can influence (and become) politicians and policy-makers, showing them that the SDGs cannot be reached without strengthening nursing [21].

Being considered dynamic professionals who respond to the needs of individuals and communities, nurses need to be prepared to be innovative in their role in the social process, developing skills to promote social, economic and political actions that not only expose inequities in the health field, but also identify innovating approaches to renovate the delivery of healthcare [22].

In this sense, understanding the context of inequities from the perspective of nurses implies assessing how these inequities are daily experienced by vulnerable populations and communities, because, even though the basic causes of inequities in the health field are structural, effects are experienced at a personal level [22]. Hence, nurses play the role of advocates, managers, leaders, caregivers, educators and researchers [16]. However, it is crucial that the nursing doctoral programs approach, with greater emphasis, the development of regional and local strategies to reduce social inequities, considering the impacts of these actions to the reduction of poverty and hunger. Thus, nursing, as the largest health workforce category, is able to contribute to studies aiming for better integration between public health policies and different social dimensions of life.

In this context, the "Nursing science and praxis: impacts on health, economy, and quality of health of individuals and groups" shows how nursing academic and professional institutions are essential for the establishment of environments conducive for the development of competencies necessary to deliver health care. Nursing doctoral programs should promote competencies such as leadership and teamwork among nurses, in addition to qualities such as professional influence and credibility, which are essential for involving nursing in global health.

Therefore, academic institutions should incorporate SDGs in their curricula, both for moral and strategic reasons [23]. Nursing scientific knowledge has the potential to improve health outcomes and advance in care technology innovation, thus, research results need to be increasingly adopted in policies and practice. Research is important to inform policy decisions, and, in that respect, nursing schools and faculties play a critical role in producing the evidence that can convince political decision-makers to invest in nursing education and in the development of the profession [24].

In this aspect, two dimensions are related to the nursing routine, knowledge and praxis, which involve the nursing care process. It is in the praxis that knowledge is perceived as one of the elements used in the exercise of the profession, enabling workers to perform their practice with competence and awareness. Hence, knowledge is incorporated in the development of techniques that constitute practical knowledge. Nonetheless, for quality care delivery that is coherent with social demands, it is crucial that workers acquire scientific knowledge, technical skills, and implement critical-reflective reasoning to satisfy those requirements.

Therefore, the training of nursing PhDs enables approximating workers with teaching at the beginning of their professional lives or after some experience with health care delivery. For those who choose to teach, technical education at the medium level represents a field of work beyond the vertical division of the nursing work, through teaching and supervision of activities and management of a care team, as well represented by the category “Education for providing necessary training to face social challenges imposed on nursing and health”.

The category “Strategies to tackling social inequities from a gender perspective: nursing and women’s empowerment" promotes reflecting upon the fact that nursing is predominantly a female choice. In this sense, the presence of women in the nursing field may contribute to combating social inequities from a gender perspective. The search for women's empowerment and defense of human rights permeates the commitment of nurses, who in the different health services and social spheres, fight for decreasing inequalities that prevent access to health services and health care [25]. There are, however, few Latin American nursing doctoral programs focusing on this topic.

The role of women in science has been discussed considering their unique contributions for the advancement of different disciplines. Nursing must increase this discussion, taking into consideration its professional profile. In several contexts, the PhD represents the only path for the achievement of leadership, enabling nurses to deal with different power and gender relationships within and outside the profession [26]. Therefore, there is a need for greater investments in research related to the development of public policies aiming at the empowerment of girls and women, especially fostering their participation in nursing, health and other decision-making spaces.

The category “Ecological systemic care: nursing ontological and epistemological bases for intervening in contexts from a sustainable perspective” prompts us to reflect upon the extent with which historical aspects of nursing influence current delivery of care. In this sense, the Environmental Theory proposed by Florence Nightingale [27], is relevant as nurses with a holistic view, are active in contexts related to environment and sustainability. Therefore, nurses are agents who design plans to cope with the consequences of climate change and potential dissemination of heart and respiratory diseases, for instance, as well as plan how to support and treat mental health in periods of drought and circumstances of fires and floods; in addition to working in situations of insanitation when houses and communities are destroyed [28, 29]. It is also possible to envision the role of nurses in contexts lacking clean drinking water, to instruct the population regarding potential diseases [30].

In this sense, the category “Labor and health: nursing implications for the healthy development of labor and workers” shows that nurses can promote dignified and fair labor, in order to favor economic growth. Hence, the training of nursing PhDs is also committed with fighting unbalances and defending systems and actions that promote opportunities for inclusive economic growth for all [31, 32].

Considering the holistic perspective of nurses, category “Technologies and innovations for nursing and health care” relates nursing and its teaching to technology. In this sense, one study [7] lists priorities of research identifying technology as a tool to be developed in order to promote innovation that can contribute to improve the competencies of nursing professors and nursing practitioners. It also can serve as a tool to impact the training of human resources in the field of nursing and to strengthen research networks to enable nurses to develop scientific studies intending to propose solutions in health.

The category, “Social nursing practice: reducing inequalities and promoting conditions for social justice” reveals that nurses play an important role in health advocacy. Global citizenship may be understood as sensitization to embrace cultural diversity and promote social fairness and sustainability, with responsible action. In this sense, it is interesting to reflect that nurses, as leaders and educators, are in a position to ensure that nurses are prepared as global citizens, to contribute to the attainment of SDGs. Therefore, concepts such as global citizenship and social justice need to be included in the curricula of nursing doctoral programs, seeking to promote social justice, equity, inclusion, and access to the right to health [31].

Considering that the greatest number of doctoral programs analyzed in this study are from Brazil, in the Brazilian context, nurses are able to defend the Unified Health System (SUS), working as advocates and ensuring human rights [33]. It is worth noting that health advocacy can promote community participation, fostering collaboration between community and health workers.

Hence, in order to achieve the SDGs, it is essential to include these topics in nursing doctoral programs, enabling nurses to play an effective role in discussions concerning public policies, as these workers are inserted in various contexts related to human life and are able to develop and direct research and practice to systemic care, in addition to being part of the largest group of health workers, having an important presence within communities [31]. Nursing doctoral programs should intend to qualify nurses for the various contexts and challenges imposed on the achievement of all SDGs, especially the ones dealing with challenges experienced in Latin America, such as poverty, lack of education, gender inequities, poor working conditions and need for an economic sustainable development.

From the 17 SDGs, five were not related in the preliminary codes: SDG 11—sustainable cities and communities; SDG 13—action to deal with climate global change; SDG 14—life below water; SDG 15—life on land and SDG 17—partnership for the goals. Based on this reality, programs must broaden their scope of possibilities considering the global impact of nursing education to regional demands. Thus, these SDGs are linked to the nursing metaparadigm, especially in issues related to environment, health, care and people.

As a limitation, the research analyzed only the PhD nursing doctoral programs which had available information on the internet regarding research areas and objectives. From the total of 56 existing programs in Latin America, this study analyzed 52 of them representing 92.8% of the total.

## Conclusion

Analysis of the 52 nursing doctoral programs in Latin America, by relating their objectives and research areas with the SDGs, revealed that most programs intend to achieve SDGs 3, Good health and well-being. Additionally, the programs are also related to SDGs 1, 2, 4, 5, 6, 8, 9, 10, 12 and 16. The SDGs 11, 13, 14, 15 and 17 were not related to any of the analyzed programs.

Thus, although nursing doctoral programs in Latin America have great potential to contribute to the training of workers with leadership skills to face the current global health challenges, there is still a need for these programs to address with more emphasis the other SDGs, especially challenges related to the reduction of social and gender inequities.

## Data Availability

Available upon request to author IACM.

## Abbreviations

SDGs: Sustainable development goals
PhD: Doctor of philosophy
SPSS: Statistical Package for the Social Sciences
